# Combination of potassium oxonate with anti-PD-1 for the treatment of colorectal cancer

**DOI:** 10.3389/fonc.2025.1528004

**Published:** 2025-02-07

**Authors:** Yuanyuan Wang, Chenxi Hu, Tianpeng Du, Jiawen Li, Kaiyuan Hui, Xiaodong Jiang

**Affiliations:** ^1^ Department of Oncology, The Affiliated Lianyungang Hospital of Xuzhou Medical University, Lianyungang, Jiangsu, China; ^2^ Department of Urology, The Second Affiliated Hospital of Nanjing Medical University, Nanjing, China

**Keywords:** anti-PD-1, CRC, immunotherapy, immune microenvironment, potassium oxonate

## Abstract

**Introduction:**

Identification of effective therapies for colorectal cancer (CRC) remains an urgent medical need, especially for the microsatellite stable (MSS) phenotype. In our previous study, potassium oxonate (PO), a uricase inhibitor commonly used for elevating uric acid in mice, unexpectedly showed remarkable inhibition of tumor growth when combined with anti-programmed death-1 (PD-1). Further research demonstrated that the combination of potassium oxonate and anti-PD-1 could reprogram the immune microenvironment. This study aimed to explore the anti-tumor effect of PO combined with anti-PD-1, and investigate the impact on the immunosuppressive tumor microenvironment (TME).

**Methods:**

We established a syngeneic mouse model of CRC and divided into groups of control group, single drugs group of PO and anti-PD-1, and the combination group. Use the HE staining, immunohistochemistry (IHC) and TUNEL staining of tumor issues to verify the anti-neoplasm of each group. We also tested the changes of TME through flow cytometry of spleen of mice in each group, as well as the IHC of cytokines.

**Results:**

The co-therapy of PO and anti-PD-1 showed admirable anti-tumor effect compared with the control group and the single drug groups. The TME were tended to an environment beneficial for killing tumors by enhancing chemotactic factor release, increasing CD8+ T cell infiltration and activation, and decreasing the amount of regulatory T cells. Moreover, IFN-γ and IL-2 secretion were found to be enriched in the tumor TME.

**Conclusion:**

Our study indicated that combination of PO and anti-PD-1 could synergistically suppress CRC progression and altered the tumor microenvironment in favor of antitumor immune responses.

## Introduction

Colorectal cancer (CRC) is the third most prevalent cancer worldwide and the leading cause of death in both men and women (1). Current statistics show that the 5-year relative survival rate for localized and distant-stage CRC is 90% and 14% (2), respectively. For patients with metastatic CRC (mCRC), treatments based on cytotoxic agents, including irinotecan, oxaliplatin, and fluorouracil, remain the first- or second-line therapy. Additionally, drugs targeting vascular endothelial growth factor (VEGF) or its receptor (VEGFR) and epidermal growth factor receptor (EGFR), such as bevacizumab, regorafenib (3), cetuximab (4), and fruquintinib (5, 6) have been approved; however, alternative therapeutic options for advanced diseases remain scarce.

In recent years, novel immunotherapy research has shown promising results for various types of cancer, including mCRC (7). Programmed death-ligand 1 (PD-L1), which is present on tumor cells (8) and/or tumor-infiltrating immune cells (IC) (9), is a classic immune checkpoint blocker (ICB); its ligation by programmed death-1 (PD-1), present on T cells, results in the inhibition of the proliferation and effector function of T cells. However, options are limited to specific molecular subtypes of mCRC because of its heterogeneous immune landscape. Therapy with anti-PD-1 or PD-L1 has resulted in remarkable success in mCRC with microsatellite instability–high phenotype (MSI-H), which has a higher tumor mutation burden (TMB), more immune infiltration (10, 11), and accounts for 3%~5% of mCRC. The microsatellite stability (MSS) type, which accounts for the remaining CRC types, has long been considered a biomarker of resistance to checkpoint inhibitors (12). The development of anti-PD-1/PD-L1 applications in mCRC is urgently needed.

Potassium oxonate (PO), an uricase inhibitor in mice, has been applied to establish a model of acute or chronic hyperuricemia with different dosages and administration methods. Research on hyperuricemia has mainly focused on gout and heart disease (13). Serum uric acid is also associated with prognosis and survival of cancers. However, few studies have investigated the effect of PO in tumors, except for determining that it is an important component of S-1, an oral fluoropyrimidine that serves to inhibit the phosphorylation of 5-FU in the gastrointestinal tract and decrease serious gastrointestinal toxicities without disrupting the antitumor effect (14, 15). S-1 is now widely used as an adjuvant and palliative chemotherapy for gastric cancer, pancreatic cancer, and CRC (16–18). In the previous study, by investigating the effect of soluble uric acid on anti-PD-1, we accidentally discovered that PO could enhance the curative effect of anti-PD-1 in CRC but not the uric acid level.

In this study, we established a syngeneic model of CRC and found that the combination of PO and anti-PD-1 synergistically promoted the antineoplastic effect of the treatment and reprogrammed the immunosuppressive TME to tumor-lethal.

## Materials and methods

### Cell culture

CT26 mouse colon carcinoma cell lines were obtained from Type Culture Collection of the Chinese Academy of Sciences (Shanghai, China). Cells were cultivated in RPMI 1640 culture medium supplemented with 10% FBS, 100 U/mL penicillin, and 100 mg/mL streptomycin and were cultured in a humidified 5% CO_2_ atmosphere at 37˚C in incubator.

### Animals

Six to eight weeks old, BALB/c mice were purchased from Wuhan Bestcell Model Bio-Tech Co., Ltd. They were housed with free access to pellet food and water in plastic cages at 21 ± 2˚C and kept on a 12-h light/dark cycle. Animal welfare and experimental procedures were carried out in accordance with the Guide for the Care and Use of Laboratory Animals (National Institutes of Health) and the related ethical regulations of Bestcell Model Biological Center. All experimental protocols were approved by the Laboratory Animal Welfare & Ethics Committee of Bestcell Model Biological Center. All efforts were made to reduce the number of animals used and to minimize animals’ suffering.

### Syngeneic model

**Figure 1:**
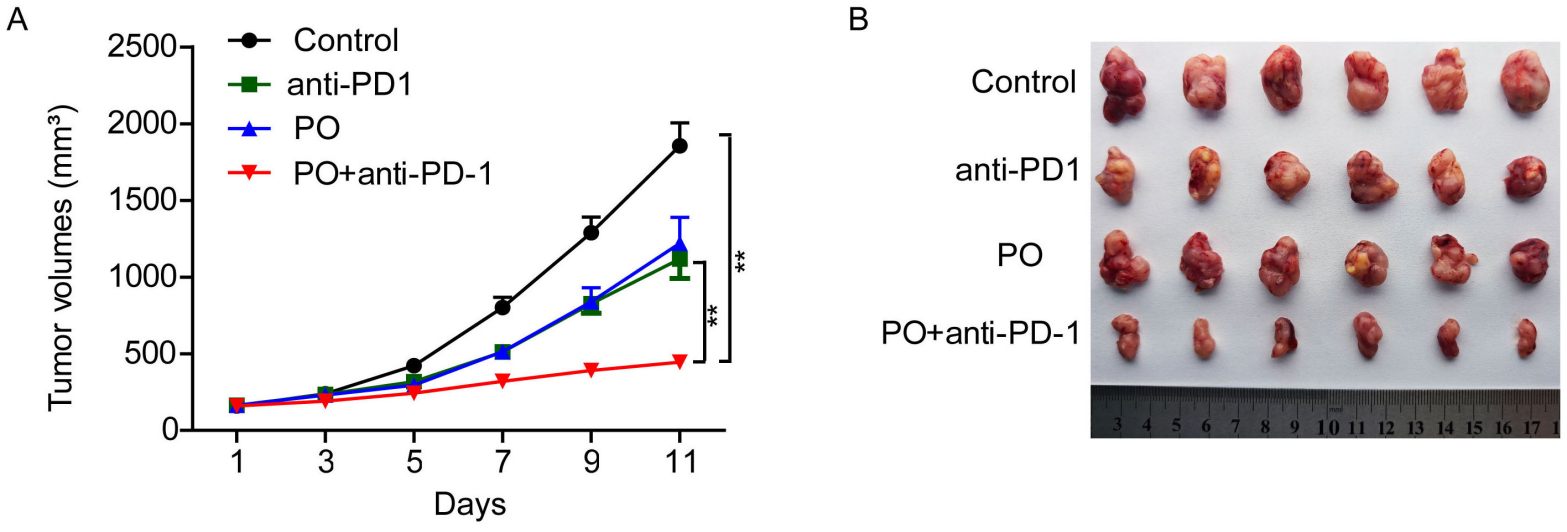
PO and anti–PD-1 cotreatment significantly inhibited tumor growth *in vivo*. CT26 cells (6×10^5^) were transplanted s.c. into the armpit of BALB/c mice. Three days after transplantation, the mice were randomly allocated to either the control or treatment groups. Drugs were given as described in Materials and Methods. **(A)** TV was measured every 2 days to the 11th day. **(B)** Solid tumors were separated after the mice were sacrificed and photographed. The data represent the mean 6 SEM of six mice per group in **(A)** to **(B)**. *p<0.05, **p<0.01, versus as indicated. ns, not significant.

CT26 cells (6×10^5^) were inoculated at the right flank of BALB/c mice (**Figure 1**), After the tumor reached 50 mm^3^, mice were randomized to four groups (n = 6 per group), and treatments were initiated as follows: group 1, mice were administered a daily oral gavage with 5% CMC.Na (vehicle); group 2, mice were administered a daily oral gavage with potassium oxonate at 250 mg/kg; group 3, mice were administered with antimouse PD-1 at 5 mg/kg by i.p. injection every 3 d; and group 4, mice were administered with potassium oxonate plus anti-mouse PD-1 Ab. Tomor volume (TV) was determined by measuring the largest diameter (a) and its perpendicular (b) according to the formula (a * b^2^)/2. The TGI (%TGI =100 * [1 - (TV _final_ -TV _initial_ for drug treated group)/(TV _final_ - TV _initial_ for control group)]) was used for evaluation of antineoplastic effect. On the 15^th^ day, mice were euthanized, and tumors were removed by scissors. The weight was measured by electronic balance in wet, and tumor sections were fixed in formalin. The rest of the sections were frozen in liquid nitrogen and stored at -80˚C.

### Immunochemistry

Immunohistochemistry was performed as previously described (19). Sections were incubated with primary antibodies overnight at 4°C, including PCNA (Boster, BM0104), IFNγ (Affinity, DF6045). Then, the sections were incubated with the secondary horseradish peroxidase conjugated antibody (Absin, Shanghai, China) for 30 minutes at room temperature. Targeted proteins were visualized with diami-nobenzidine (Zhongshan Golden Bridge, Beijing, China). The results of IHC were determined by staining intensity and the number of positive cells.

### TUNEL assay

Paraffin-embedded tumor sections were stained with TUNEL Detection Kit (G1504, Servicebio, Wuhan) and then counter-stained with DAPI for 20~30min. Images were acquired using Eclipse Ci-L (Nikon).

### Flow cytometry assay

Spleen tissues from CRC model were harvested on the days indicated. To obtain single-cell suspensions, the spleen was first scraped with scissors, and the homogenate was then passed through a 0.45 μm nylon mesh and and washed twice with PBS/0.5% BSA. Single-cell suspensions were stained with surface mAb: FITC-conjugated anti-CD4 (Invitrogen, 11-0041-81), FITC-conjugated CD8 (Invitrogen, 11-0081-81), and PE-conjugated CD25 (Invitrogen, 12-0251-81) for 30 minutes at 4°C. For the intracellular markers, cells were incubated in 24-well, flat-bottom plates with cell stimulation mixture (eBioscience/Thermo Fisher Scientific, Waltham, MA) for 4 h under a humidified 5% CO2 atmosphere at 37˚C in incubator and fixed and permeabilized with the FoxP3/Transcription Factor Staining Buffer Set (eBioscience/Thermo Fisher Scientific) according to the manufacturers’ protocols, then stained with intracellular markers: APC-conjugated Foxp3 (Invitrogen, 17-5773-80). Samples were collected on a CytoFLEX Flow Cytometer (Beckman), and data were analyzed with FlowJo.

### Statistical analysis

GraphPad Prism 5.0 (La Jolla, CA, USA) was used to analyze the data, which are expressed as mean ± SEM. Statistical significance (*p < 0.05, **p < 0.01, ***p, 0.001) was assessed using two-tailed unpaired Student’s t-tests for comparisons between two groups.

## Results

### Combined PO and anti-PD-1 significantly inhibited tumor growth in a syngeneic model of CRC

To investigate whether PO plus anti-PD-1 exerts synergistic antineoplastic effect *in vivo*, CT26 mouse colon carcinoma cells were s.c. transplanted to establish syngeneic murine models. As a result, we observed little inhibition of tumor growth in mice treated with anti-PD-1 (TV: 1118.62 ± 127.33 mm^3^) or PO (TV: 1218.77 ± 169.35 mm^3^) alone compared to that in mice treated with vehicle (0.5% CMC.Na; TV: 1854.25 ± 150.20 mm^3^), with tumor growth inhibitions of 40% and 35.3%, respectively. In contrast, the combination therapy group showed remarkable inhibition of tumor growth. On the day of sacrifice, the TV was 445.2 ± 23.2 mm^3^, while the TGI was 76% (**Figures 1A, B**). Thus, this finding inferred that PO plus anti-PD-1 inhibit the tumor growth.

### PO plus anti-PD-1 antibody inhibited proliferation and induced tumor cell apoptosis *in vivo*

**Figure 2:**
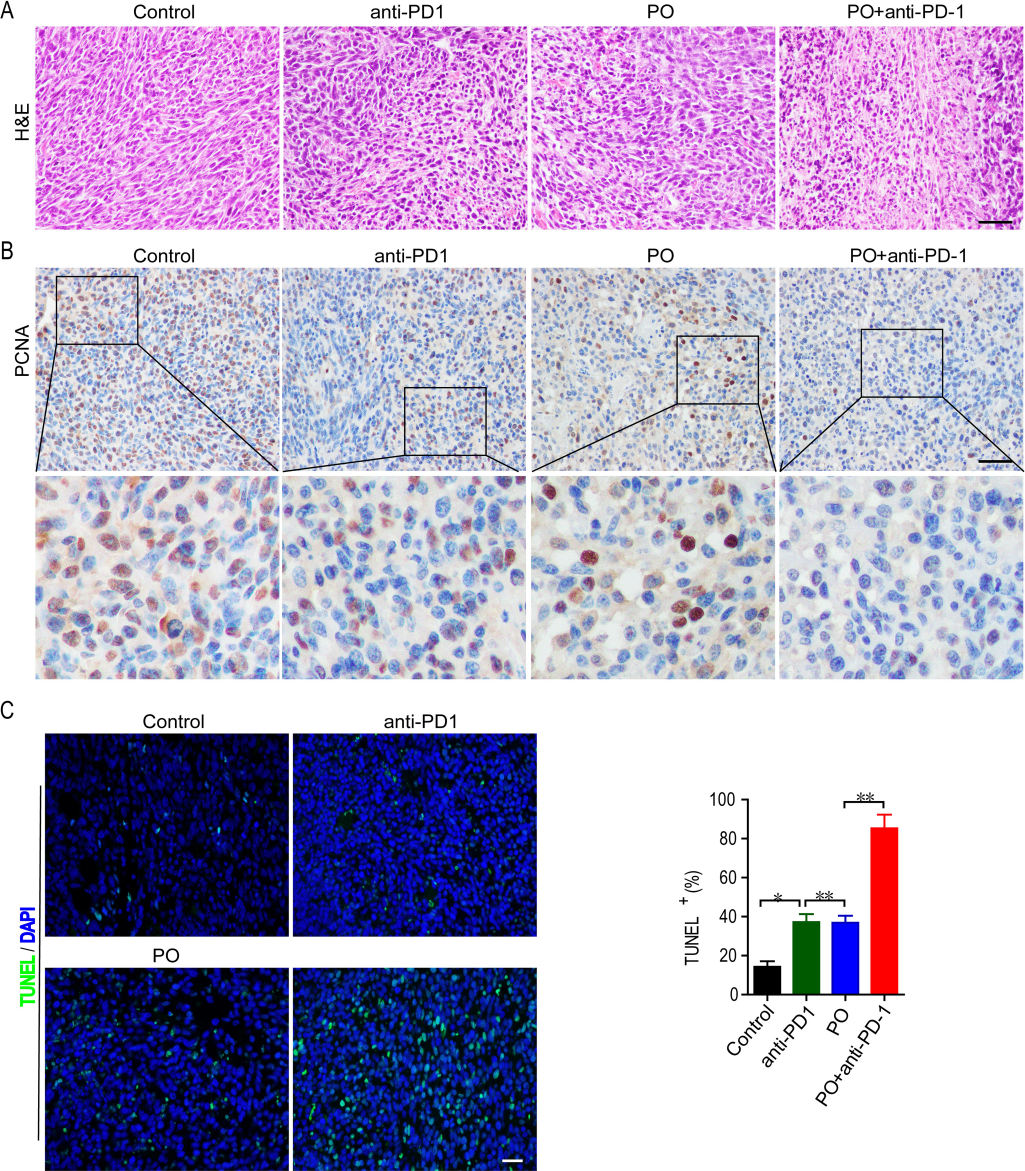
Combination of PO and PD-1 blockade inhibited proliferation and induced apoptosis of tumor *in vivo*. **(A)** Paraffin sections of CT26 tumor tissues were analyzed by H&E staining (n = 3). **(B)** Expression and quantification of PCNA-positive staining in CT26 tumor tissues was examined by IHC using Image-Pro Plus 6.0 and in three random fields (n = 3). Scale bar, 50 μm. **(C)** TUNEL staining and the quantification of TUNEL-positive cells in CT26 tumor tissues (n = 3). Scale bar, 20 μm *p < 0.05, **p < 0.01, versus as indicated. ns, not significant.

We further observed the effects of PO plus anti-PD-1 on the proliferation of tumor cells *in vivo*. Histological analyses by H&E staining showed that PO with anti-PD-1 strongly induced massive amounts of cell damage, with nuclear shrinkage, sparse arrangement, and fragmentation of tumor cells (**Figure 2A**). Next, the proliferation and apoptosis of tumor tissues in each group were examined to confirm the inhibitory effect of the combination of PO and anti-PD-1. Consistent with the results of H&E staining, immunohistochemistry of PCNA showed a sharp decrease in the expression of PCNA protein in the tumor tissues of the combined treatment group compared to that in the single-drug treated group (**Figure 2B**). TUNEL staining confirmed that PO combined with anti-PD-1 triggered extensive tumor cell apoptosis (**Figure 2C**). These results demonstrated that co-treatment with PO and anti-PD-1 led to elevated inhibition of proliferation and induction of tumor cell apoptosis *in vivo*.

### PO plus anti-PD-1 reduced regulatory T cells and elevated CD8+ T cells in the spleen

**Figure 3:**
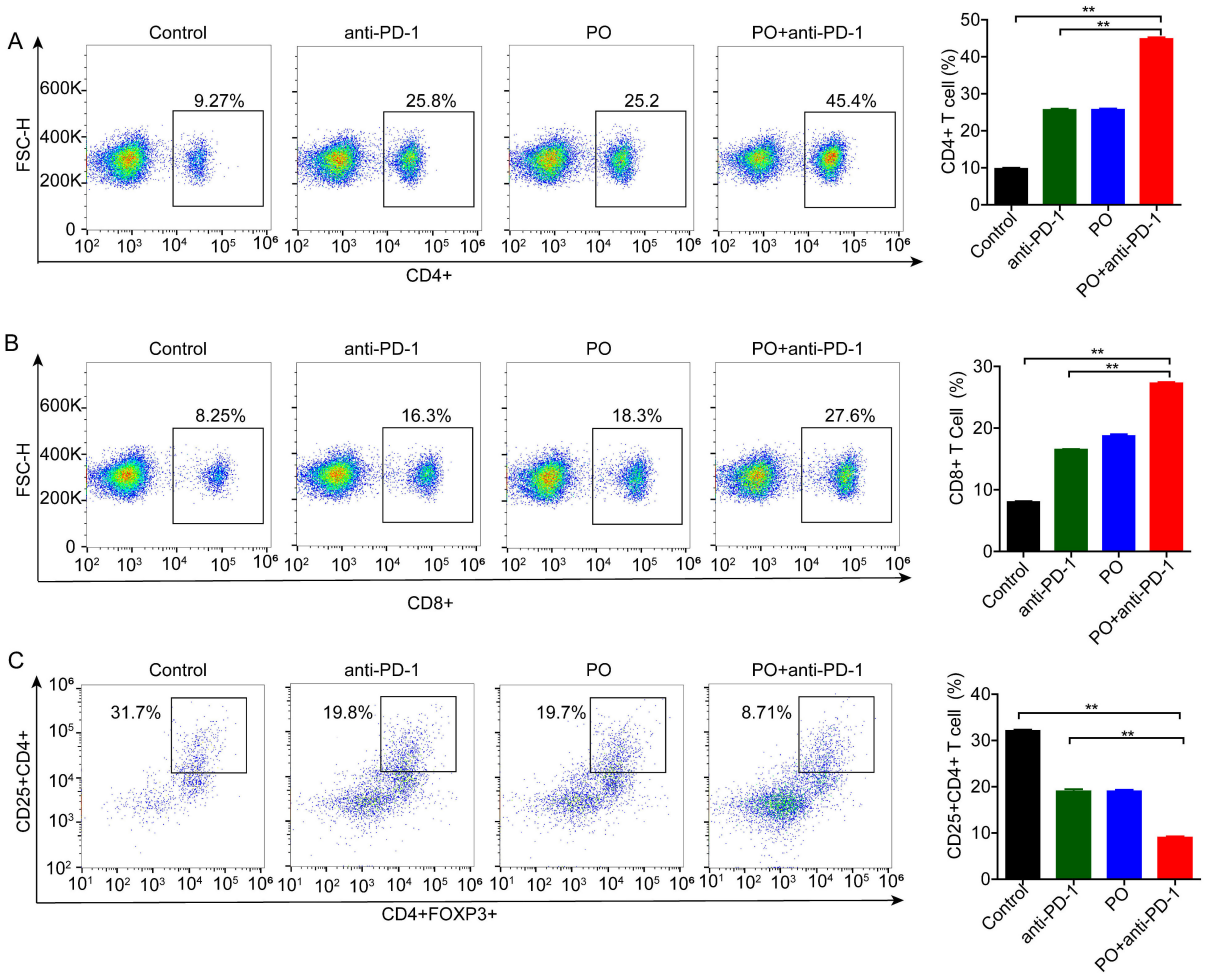
Combination of PO with anti-PD-1 blockade substantially reduced regulatory T (Treg) cells but activated CD4^+^ and CD8^+^ cells. Splenic T cells from CT26-bearing mice were collected and subjected for intracellular staining for Treg cells. **(A)** Representative images of the gating strategy to define CD45^+^CD4^+^Foxp3^+^ Treg cells in each group. **(B, C)** Percentages of CD4^+^ **(B)** and CD8^+^ **(C)** T cells. The data represent the mean ± SEM of three mice per group, *p < 0.05, **p < 0.01 versus as indicated. ns, not significant.

The efficacy of anti-PD-1 is intimately associated with immune cell infiltration and function in the TME. To test whether changes in Treg cells occur in the periphery, we detected immune cells in the spleen using flow cytometry. The results showed that monotherapy reduced the differentiation of Treg cells (by 12.9%), and this effect was further enhanced by co-therapy (by a further 11.0%) (**Figure 3C**). We subsequently analyzed the activation of antitumor lymphocytes. Compared to the control, PO plus anti-PD-1 increased both the proportion of CD4^+^ (**Figure 3A**) and CD8^+^ T cells (**Figure 3B**). These results suggest that the combination of PO and anti-PD-1 augmented the antitumor immune response by reducing Treg cells and enhancing the infiltration of CD8^+^ T lymphocytes.

### PO plus anti-PD-1 antibody promoted T cell infiltration and function

**Figure 4:**
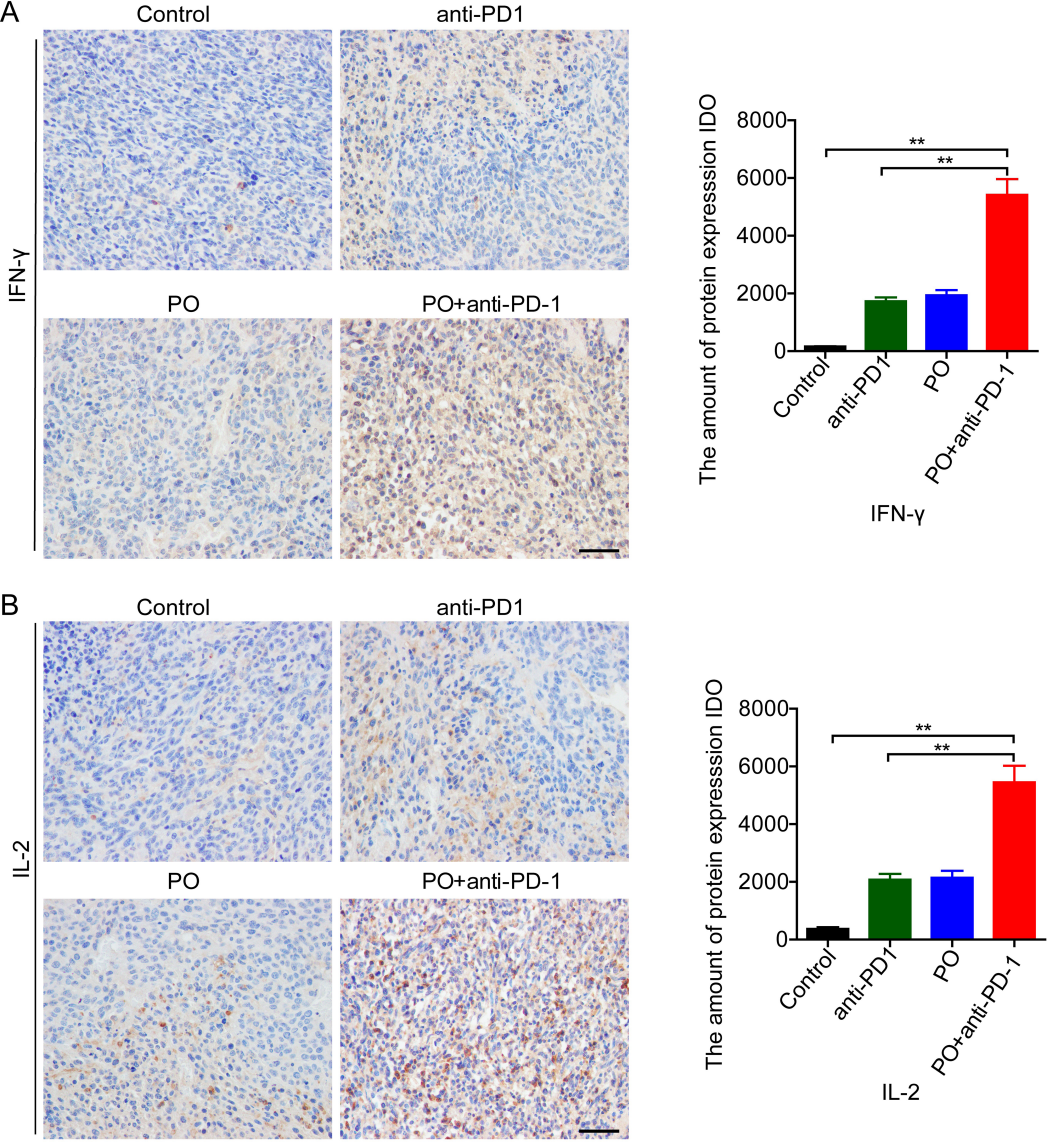
PO plus anti–PD-1 promoted antitumor cytokines in tumor tissue **(A, B)** IFN-γ **(A)** and IL-2 **(B)** expression in CT26 tumor tissue were examined by IHC and quatilized by integral optical density (IDO). Scale bar, 50 μm. The data represent the mean ± SEM of three mice per group. *p < 0.05, **p < 0.01, versus as indicated. ns, not significant.

Successful anti-PD-1 cancer immunotherapy requires T cell-dendritic cell crosstalk involving the cytokines IFN-γ and IL-12 (20). Further study indicated that IL-2 delivery by engineered mesenchymal stem cells re-invigorate CD8+ T cells to overcome immunotherapy resistance in cancer (21). In our study, PO combined with anti-PD-1 greatly enhanced the secretion of IFN-γ (**Figure 4A**) and IL-12 (**Figure 4B**) compared to the control group and the anti-PD-1 or PO single drug. These results indicated that PO plus anti-PD-1 elevated antitumor immune activity in tumors, thus resulting in a promising antitumor immune microenvironment.

### PO caused no damage to normal organs

**Figure 5:**
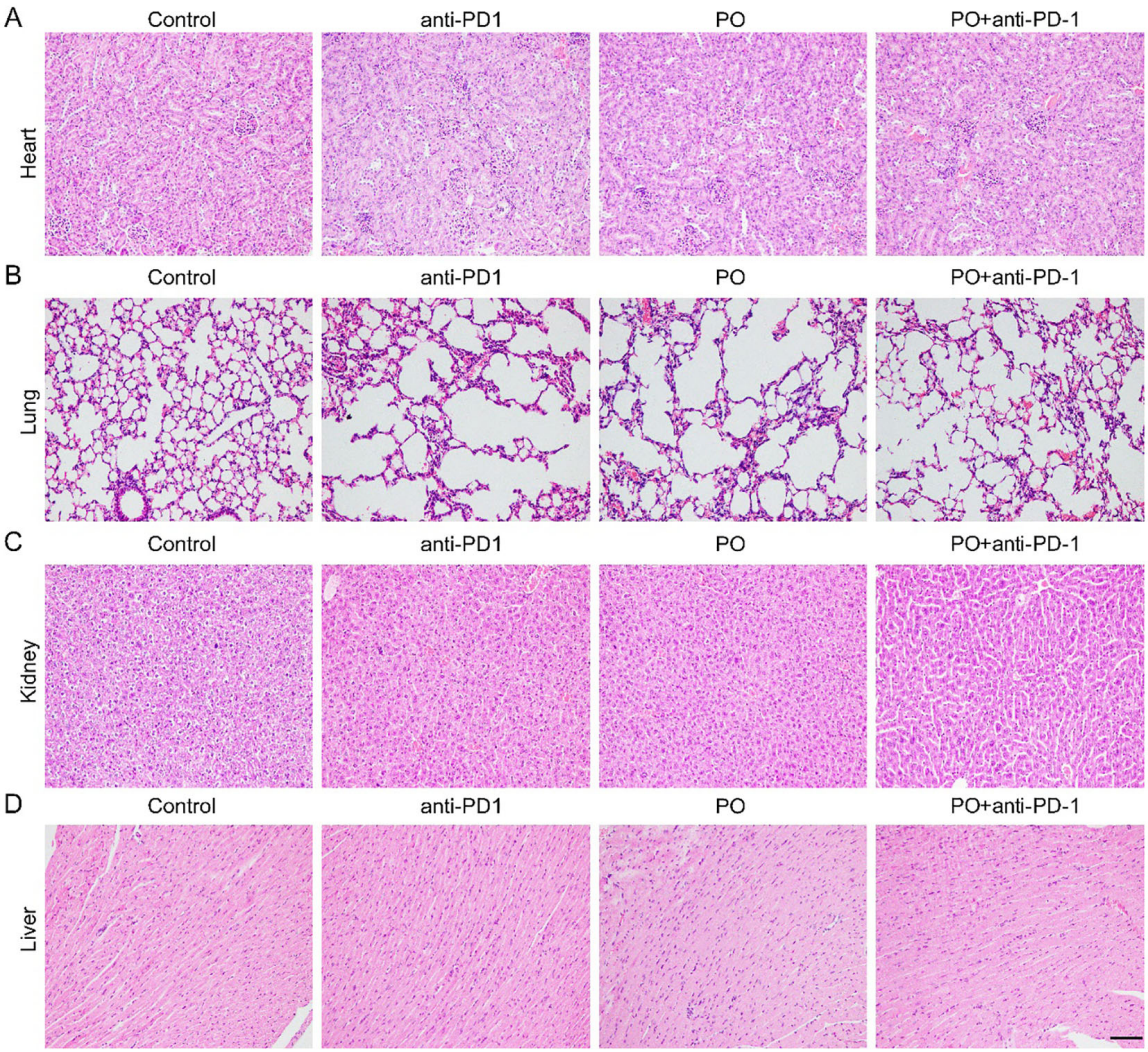
PO and/or anti–PD-1 had no damage to normal organs. Paraffin sections of normal organs in CT26 tissues were analyzed by H&E staining. Scale bar, 50 μm. **(A-D)**. H&E staining of heart **(A)**, lung **(B)**, kidney **(C)**, liver **(D)** tissues in four groups ( n =3).

The applications of PO usually cause hyperuricemia, which is an overlooked cardiovascular and renal risk factor. Epidemiological and genetic studies have shown an independent role of uric acid in the risk of coronary artery disease, heart failure, chronic kidney disease, and cardiovascular mortality (22). In our study, we excised the main organs, including the heart, lung, kidney, and liver at the sacrifice of mice (**Figures 5A–D**). Histological analyses by H&E staining revealed that all of the organs maintained a normal cell morphology and skeletal structure in both the control and treatment groups, for example, the structure of alveolus in lung were well-formed and cell morphology in heart, kidney and liver showed pretty good shape, which indicating no damage. Thereby, the reliable safety of short-term and low-dose PO gavage.

## Discussion

Single agent treatment with anti-PD-1 monoclonal antibody significantly prolongs median PFS in patients with advanced MSI-H/dMMR phenotype of colorectal cancer. In the KEYNOTE-177 study, pembrolizumab monotherapy extended the median PFS compared to chemotherapy and/or targeted therapy (16.5 months vs 8.2 months). But the vast majority of colorectal tumors are MSS/pMMR phenotype, a typical “cold tumor” (23), which is not sensitive to single anti-PD-1/PD-L1 monoclonal antibody therapy. Thus, identifying optimal combinatorial strategies to enhance the efficacy of anti-PD-1/PD-L1–based immunotherapy is important research to undertake to combat MSS CRC.

Factors of invalid anti-PD-1/PD-L1 therapy in “cold cancers” include low tumor mutational burden, poor intrinsic antigenicity of tumor cells, defective antigen presentation, exhausted T cell functions, and tumor suppression-associated macrophage accumulation (24). Immunosuppressive metabolic pathways have become research hotspots in recent years. For example, the ratio of adenosine and indoleamine-2,3-dioxygenase accounts for the curative effect of anti-PD-1/PD-L1 therapy (25). To overcome the antitumor effect, many clinical trials have sought to establish combinations of anti-PD-1 and other therapies, such as chemotherapy (26), targeted therapy (27), radiotherapy (28), or other immunotherapies (12). The latest research indicated that the combination of a PD-1 antibody, a histone deacetylase inhibitor (HDACi), and a VEGF antibody could be a promising treatment regimen for patients with MSS/pMMR advanced CRC (29). Nevertheless, some patients still cannot tolerate the side effects of chemotherapy or other treatments in combination therapy, leading to treatment interruption. In this study, we originally attempted to discover the impact of soluble uric acid in peripheral blood on the effects of anti-PD-1/PD-L1 therapy in CRC by constructing a high uric acid tumor-bearing mouse model. Although no correlation was found, following careful analysis of the experimental data, we surprisingly discovered that PO (250 mg/kg) plus anti-PD-1 effectively inhibited tumor growth. The mechanism of the curative effect of such combination treatment may involve the elimination of drug resistance to anti-PD-1 by PO or a synergistic improvement of the efficacy of the two drugs. Indeed, it was reported that PO had a limited antitumor effect.

PO, with a molecular formula of C_4_H_2_KN_3_O_4_, was first found as an inhibitor of uricase to induce hyperuricemia in mice and rats because they synthesize the uricase enzyme to metabolize uric acid to allantoin, which is different in humans and great apes. In hyperuricemia animal models, PO tends to be given through oral gavage or intraperitoneally injected at 250 mg to 1000 mg/kg for 5 days to 8 weeks (30). Uric acid is related to the occurrence, development, and treatment of many diseases, including renal dysfunction, gout, leukemia, coronary heart disease, and so on; therefore, mice with hyperuricemia often experience corresponding tissue damage, which is either short term or long term. Another application of PO is as a gastrointestinal protective agent to reduce the injury of GI tissues or severe diarrhea caused by 5-FU treatment, without a decline in antitumor effect. Whether oral gavage of S-1 or intravenous injection of PO and its metabolites in mice, PO is mainly converted to melamine in the gastrointestinal tract (31). Even though PO is metabolized in the gastrointestinal tract, its concentration in intestinal cells is much higher than that of 5-FU. One possible mechanism of this is that PO (10~50 mg/kg) competitively inhibits orotate phosphoribosyltransferase and decreases the levels of 5-fluorouridine-5’-monophosphate (FUMP) and 5-FU incorporated into RNA by approximately 70% in the small intestine, as compared to only 0%~20% in bone marrow and tumors (31). Tetsuro Yamashita et al. found that PO could inhibit the anticancer-drug-induced decrease in NK activity and maintain IL-2 production by lymphocytes stimulated by tumor antigens in cancer-bearing rats (14). Moreover, the maintenance of IL-2 production by PO may act to preserve antitumor immunity, without disturbing the induction of cytotoxic T cells *in vivo*. However, this study compared the S-1 group with the tegafur (FT) + 5-chloro-2, 4-dihydroxypyridine (CDHP) group, while the PO group was absent. Moreover, as the changes in immune cells and cytokines were only analyzed in the spleen and mesenteric lymph nodes, but not in tumor tissues, no direct antitumor research of PO was conducted (32). In our study, we set up a PO single drug group with 250 mg/kg through oral gavage daily for 15 days. The results showed a limited anti-tumor effect compared to that observed in the control group and no obvious impact on antitumor immunity. Moreover, no obvious damage was observed from the H&E staining of major organs, which seemed to confirm the safety of PO at 250 mg/kg. Surprisingly, PO combined with anti-PD-1 greatly inhibited the growth of CRC, potentially indicating a unique role in anti-tumor therapy. Anti-PD-1 has been widely used in various cancers (33), while PO seems to serve as a sensitizer for anti-PD-1 in CRC curation. Thus, more low doses of PO should be verified. However, we could not rule out the impact of elevated uric acid on the efficacy of anti-PD-1. PO may, through high level of uric acid to enhance the anti-plastic potency when combined with anti-PD-1.

The TME generates an immunosuppressive niche that limits the expansion and function of tumor-infiltrating lymphocytes (TILs) and eventually fails to respond to immunotherapy in the late stage, while cytokines that have no anti-tumor effect can fill this immunosuppressive niche (34). Clinical studies consistently support the view that MSS-type mCRCs are nonpermissive to T effector cell accumulation and are usually infiltrated with abundant immune suppressors, such as tumor-associated macrophages, myeloid-derived suppressor cells, and Treg cells (35). Thus, only overcoming these obstacles can break ICB resistance and elicit anti-tumor immunity. In our study, PO played a critical role in regulating key antitumor immune responses through mechanisms such as suppression of Treg cell proliferation and enhancement of antitumor lymphocyte infiltration and function, both of which were further enhanced by anti-PD-1. We also observed that the expression of specific chemokines, such as IFN-γ and IL2, was significantly increased in the combination treatment CRC group and identified potential biomarkers. Several studies have suggested that exogenous IL-2 in the TME activates and expands pre-existing CD8^+^ TILs (21), while the immune cytokine L19-IL2 combined with single-dose RT resulted in 75% tumor remission and a 20% curative abscopal effect in the T cell-inflamed C51 CRC model (36). However, IFN-γ could drive PD-L1 immunosuppression (37) and sustained type I interferon signaling is a mechanism of resistance to PD-1 blockade (24). Further, in solid cancers, the surface expression of chemokine receptors on activated T lymphocytes does not always match the cognate ligand expression at the tumor site.

In conclusion, the current study is a proof of concept that PO, in combination with anti-PD-1, shows an enhanced therapeutic effect in CRC models by optimizing the antitumor microenvironment that promoted an immunopermissive microenvironment. Our results also indicate that the combination of PO and anti-PD-1, which has rarely been tested in preclinical or clinical studies, may be sufficient to appropriately reprogram the immune microenvironment and enhance immunotherapy efficacy. These findings prompt future studies of this combination therapy in CRC or other cancers, which may represent a potential strategy to broaden the benefit of anti-PD-1/PD-L1 treatment, especially for those who inability to tolerate chemotherapy or targeted therapy. Future studies should establish the extent of the sustained beneficial and adverse effects of long-term administration of PO plus anti-PD-1, especially in humanised or clinical models. The direct mechanism by which PO serves to increase the efficacy of PD-1 monoclonal antibody remains to be elucidated. Potential target drugs should avoid hyperuricemia or other harmful effects of PO. Our findings should be validated using patient-derived tumor xenograft models and biological markers with promising efficacy in the future.

## Data Availability

The original contributions presented in the study are included in the article/supplementary material. Further inquiries can be directed to the corresponding authors.
